# Intratracheal transplantation of trophoblast stem cells attenuates acute lung injury in mice

**DOI:** 10.1186/s13287-021-02550-z

**Published:** 2021-08-30

**Authors:** Junwen Han, Gu Li, Minmin Hou, Julie Ng, Min-Young Kwon, Kevin Xiong, Xiaoliang Liang, Elizabeth Taglauer, Yuanyuan Shi, S. Alex Mitsialis, Stella Kourembanas, Souheil El-Chemaly, James A. Lederer, Ivan O. Rosas, Mark A. Perrella, Xiaoli Liu

**Affiliations:** 1 Division of Pulmonary and Critical Care Medicine, Department of Medicine, Brigham and Women’s Hospital, 75 Francis Street, Boston, MA 02115 USA; 2 Department of Medicine, Division of Pulmonary, Critical Care and Sleep Medicine, Baylor College of Medicine, Houston, TX 77024 USA; 3 Department of Pediatrics, Division of Newborn Medicine, Boston Children’s Hospital, Boston, MA 02115 USA; 4 School of Life Sciences, Beijing University of Chinese Medicine, Beijing, 100029 China; 5 Department of Surgery, Brigham and Women’s Hospital, Boston, MA 02115 USA; 6 Department of Pediatric Newborn Medicine, Brigham and Women’s Hospital, Boston, MA 02115 USA

**Keywords:** Trophoblast stem cells, Acute lung injury, Inflammation, Alveolar epithelial cells, Engraftment

## Abstract

**Background:**

Acute lung injury (ALI) is a common lung disorder that affects millions of people every year. The infiltration of inflammatory cells into the lungs and death of the alveolar epithelial cells are key factors to trigger a pathological cascade. Trophoblast stem cells (TSCs) are immune privileged, and demonstrate the capability of self-renewal and multipotency with differentiation into three germ layers. We hypothesized that intratracheal transplantation of TSCs may alleviate ALI.

**Methods:**

ALI was induced by intratracheal delivery of bleomycin (BLM) in mice. After exposure to BLM, pre-labeled TSCs or fibroblasts (FBs) were intratracheally administered into the lungs. Analyses of the lungs were performed for inflammatory infiltrates, cell apoptosis, and engraftment of TSCs. Pro-inflammatory cytokines/chemokines of lung tissue and in bronchoalveolar lavage fluid (BALF) were also assessed.

**Results:**

The lungs displayed a reduction in cellularity, with decreased CD45^+^ cells, and less thickening of the alveolar walls in ALI mice that received TSCs compared with ALI mice receiving PBS or FBs. TSCs decreased infiltration of neutrophils and macrophages, and the expression of interleukin (IL) 6, monocyte chemoattractant protein-1 (MCP-1) and keratinocyte-derived chemokine (KC) in the injured lungs. The levels of inflammatory cytokines in BALF, particularly IL-6, were decreased in ALI mice receiving TSCs, compared to ALI mice that received PBS or FBs. TSCs also significantly reduced BLM-induced apoptosis of alveolar epithelial cells in vitro and in vivo. Transplanted TSCs integrated into the alveolar walls and expressed aquaporin 5 and prosurfactant protein C, markers for alveolar epithelial type I and II cells, respectively.

**Conclusion:**

Intratracheal transplantation of TSCs into the lungs of mice after acute exposure to BLM reduced pulmonary inflammation and cell death. Furthermore, TSCs engrafted into the alveolar walls to form alveolar epithelial type I and II cells. These data support the use of TSCs for the treatment of ALI.

**Supplementary Information:**

The online version contains supplementary material available at 10.1186/s13287-021-02550-z.

## Background

Acute lung injury (ALI) is a common and devastating respiratory disorder, with an annual incidence of 86.2 per 100,000 people, and the hospital mortality rate for all patients is close to 40% [1, 2]. There are 190,600 acute cases in the USA each year, which is associated with 74,500 deaths and 3.6 million hospital days [1, 2]. In patients who survive ALI, their long-term quality of life is severely affected. Thus, there is an unmet need for better treatment options for patients with ALI [1, 2]. The causes of ALI are diverse, including lung infections of various pathogens, sepsis, harmful chemicals, and antineoplastic therapies. One such therapeutic agent is bleomycin (BLM), which is used to treat various malignancies. However, a limitation of BLM use clinically is pulmonary toxicity [3]. BLM causes its toxic effect on cells by binding with oxygen and divalent metals to produce reactive oxygen species, resulting in damage to the lung parenchyma [3]. Administration of BLM intratracheally in experimental models results in an initial site of injury in the airway epithelium [4], while systemic administration causes more endothelial injury. The histopathological features of ALI in patients and animal models indicate that inflammatory cells infiltrate into the lungs, causing damage to the alveolar-capillary barrier due to the death of the alveolar epithelial type I/II and endothelial cells, which eventually causes impaired gas exchange, hypoxemia, and the development of acute respiratory distress syndrome (ARDS) [1, 2]. Finally, patients with unresolved ARDS may progress to a fibrotic phase of the disease [5], while BLM administration in mice also evolves from acute lung injury to lung fibrosis [6]. Thus, BLM-induced experimental lung injury, including the more acute injury response, has relevance for investigation.

Transplantation of stem cells has been applied to treat ALI in humans and various animal models [7–11]. The targeted goals of stem cell therapy in ALI include regulating the inflammatory response, reducing injury, and repairing damaged tissue. Mesenchymal stem/stromal cells (MSCs) have been demonstrated to reduce the inflammatory response in experimental ALI [12], and to be safe when administered to patients with ARDS [11]. Experimental observations of endothelial progenitor cells [13], embryonic stem cells (ESCs) and induced pluripotent stem cells (iPSCs) in the treatment of ALI have been shown to regenerate epithelial cells in ALI as well [9, 10]. However, there are still challenges in the application of stem cells, especially regarding resources, ethical concerns, potential of tumorigenesis, and rejection of the allograft cells.

The placenta is readily available at the time of birth, thus there are no ethical concerns. The placenta contains a variety of stem cells [14–17] that can be easily obtained and banked. One subpopulation of placenta-derived cells, trophoblast stem cells (TSCs) are a unique population of stem cells that originate from the fetal portion of the placenta and have been shown to differentiate into other types of trophoblast cells [18], as well as the three germ layers of the fetus [19]. In addition, these cells have no expression of classical major histocompatibility complexes (HMC) class I and class II [19], decreasing the concern of transplant rejection of allogeneic cells. Interestingly, TSCs have been reported to be less risk in tumorigenesis compared with ESCs and iPSCs [20]. Thus, TSCs are drawing attention as a promising therapeutic strategy for allogeneic stem cell transplantation. To our knowledge, no investigation of administering TSCs during experimental ALI has been reported. The present study focuses on intratracheal delivery of TSCs, derived from near term placentas (embryonic day 18.5), in BLM-induced ALI. The effect of TSCs in ALI was assessed for tissue inflammation, alveolar cell injury/death, and engraftment of TSCs during the acute phase of BLM exposure. We demonstrate for the first time that TSCs play an important role in mediating tissue inflammation, preventing lung injury, and engrafting and differentiating into alveolar epithelial cells in this experimental model of ALI in mice.

## Materials and methods

### BLM-induced ALI in mice

Male, 8–10 week-old C57BL/6 mice (*n* = 51) were randomly divided into two groups. Intratracheal delivery of PBS (sham, *n* = 11) or BLM (ALI, *n* = 40) was performed as previously described [10, 21]. Briefly, 100 μL of PBS or BLM (Bleomycin Sulfate, Cayman Chemical) diluted in 100 μL PBS at a concentration of 0.75 mg/kg was intratracheally injected into the lungs of mice [21]. The use of mice and the studies performed were carried out in accordance with the Public Health Service policy on the humane care and use of laboratory animals, and the protocol was approved by the Institutional Animal Care and Use Committee of Brigham and Women’s Hospital.

### Cell preparation and delivery into mice

TSCs were harvested as previously described [19]. Briefly, placentas were collected at embryonic day (E) 18.5 from pregnancies of wild-type C57BL/6 mice and dissociated in an enzyme buffer. The cells were cultured in DMEM/F12 (Lonza) supplemented with 20% fetal bovine serum (FBS, GE Healthcare), 100 mM nonessential amino acids (Cellgro), 55 mM beta-mercaptoethanol (Gibco), 1 mM sodium pyruvate (Cellgro), 10 ng/mL leukemia inhibitory factor (Millipore), 20 ng/mL murine basic fibroblast growth factor (PeproTech), and 1% penicillin/streptomycin/l-glutamine (Corning). CD117^+^ TSCs were isolated using anti-mouse CD117 MicroBeads (Miltenyi Biotec). The clonal CD117^+^ TSCs were obtained from a limiting dilution culture and then harvested using cloning cylinders and expanded in culture for all experiments.

Fibroblasts (FBs) were harvested as previously described [22]. Briefly, the lungs were harvested from wild-type C57/BL6 mice, minced and incubated in 0.1% collagenase I and 0.25% collagenase II. The cells were subsequently isolated by lineage negative/Sca1-depleted beads to exclude contamination of fibroblasts with lung MSCs (Miltenyi Biotec, Auburn, CA). Passage 3 FBs were used for experiments. FBs were characterized by the expression of some mesenchymal markers, such as CD140b, CD105, CD44, and CD29. However, they did not express CD73, and there was no evidence for expression of CD45, a marker of the leukocyte lineage of hematopoietic cells.

Also, the cells harvested from the lungs TSCs and FBs were pre-labeled with PKH67 (Sigma), as previously described [19]. At day 3 after exposure to BLM, ALI mice were divided into three groups, receiving intratracheal administration of 100 μL PBS, FBs (0.5 × 10^6^ cells in 100 μL PBS), or TSCs 0.5 × 10^6^ cells in 100 μL PBS). Sham mice received 100µL of PBS intratracheally.

### Evaluation of bronchoalveolar lavage fluid (BALF)

A total of 600 to 800μL of BALF was collected as previously described [10, 21] for cell counts, and differential analysis of neutrophils and macrophages.

Undiluted samples (20 mL) were used for analysis by Luminex FlexMap 3D assay system per manufacturer’s instructions [23]. The levels of interleukin (IL) 6, monocyte chemoattractant protein (MCP) 1, and keratinocytes-derived chemokine (KC) were determined by standard curve analysis.

### Immunohistochemistry staining

The left lungs of mice were harvested on days 7 and 14 after BLM exposure. Tissue was fixed with 10% formaldehyde as previously described [19]. MLE-12 cells were fixed with 4% paraformaldehyde, and permeabilized with 0.2% Triton X-100. Samples were incubated with primary antibody: Aquaporin 5 (AQP5, EMD Milipore) or Prosurfactant Protein C (SPC, Abcam), at 4 °C overnight, following by second antibodies conjugated with fluorescence, at 37 °C for 1 h and nuclei staining with 4′,6-diamidino-2-phenylindole (DAPI) at 37 °C for 10 min. Staining of the lungs was also performed for Hematoxylin and Eosin (H&E), and CD45 (BD Biosciences). The lung sections were next stained to assess apoptosis with terminal deoxynucleotidyl transferase dUTP nick end labeling (TUNEL), as descripted previously [24].

### Injury and inflammatory mediators of MLE-12 cells exposed to BLM in vitro

MLE-12 (ATCC) were seeded at 1 × 10^6^ cells per 60-mm dish [24] and cultured in medium supplemented with 2% FBS, 0.005 mg/mL insulin (Sigma), 0.01 mg/mL Transferrin (Sigma), 30 nM Sodium selenite (Sigma), 10 nM Hydrocortisone (Sigma), 10 nM Beta-estradiol (Sigma), 10 mM HEPES (FisherBiotech), overnight. The cells were treated with PBS or BLM (50 µg/mL) for 24 h and then co-cultured with or without TSCs for 16 h. The cells were collected for staining using the FITC Annexin V Apoptosis Detection kit (BD Pharmingen), following the manufacturer’s protocol, and then flow cytometry assessment. To assess the regulation of inflammatory mediators by exposure of lung epithelial cells to BLM-injury, MLE-12 cells were treated with PBS, BLM (50 µg/mL) or BLM plus TSCs conditioned medium (TSC-CM) for 24 h, and then the cells were harvested for total RNA extraction. qRT-PCR was performed for inflammatory assessment.

### Quantitative real-time PCR (qRT-PCR)

Total RNA was extracted from the right two lobes of the lungs using Trizol reagent (Invitrogen) or MLE-12 cells. SuperScript III First-Strand Synthesis System was used for generation of cDNA, and qRT-PC was performed using the Syber Green kit (Bio-Rad). IL-6: 5′-CAAAGCCAGAGTCCTTCAGAG-3′, 5′-GTCCTTAGCCACTCCTTC-3′; MCP-1: 5′-GCTCTCCAGCCTACTCATTG-3′, 5′-GTCCCTGTCATGCTTCTGG-3′; KC: 5′-CAGACGGTGCCATCAGAG-3′, 5′-AACCGAAGTCATAGCCACAC-3′. AQP5: 5′-CTCCCCAGCCTTATCCATTG-3′, 5′-CCAGAAGACCCAGTGAGAGG-3′; SPC: 5′-CAAACGCCTTCTCATCGTGGTTGT-3′, 5′-TTTCTGAGTTTCCGGTGCTCCGAT-3′.

### Flow cytometry analysis for TSC engraftment

Two lobes of the right lung were digested using collagenases, as previously described [19]. An unstained cell suspension was used for an assessment of percentage of green cells in the lung. The remaining cells were fixed, permeabilized, blocked, and then incubated with primary antibodies AQP5 (EMD Millipore) and SPC (Abcam) at 4 °C for 30 min in darkness, and then the secondary antibody, at the same concentration as the primary antibodies, conjugated with fluorescence for 30 min on ice. The cells were then assessed using BD FACS Canto II and the data was analyzed using FlowJo software.

### Statistics

One-way analysis of variance was performed when more than two groups were analyzed. For comparisons between two groups, we used Student’s unpaired *t* test. Statistical significance was accepted at *P* < 0.05.

## Results

### Transplantation of TSCs attenuates inflammation in ALI

**Fig. 1:**
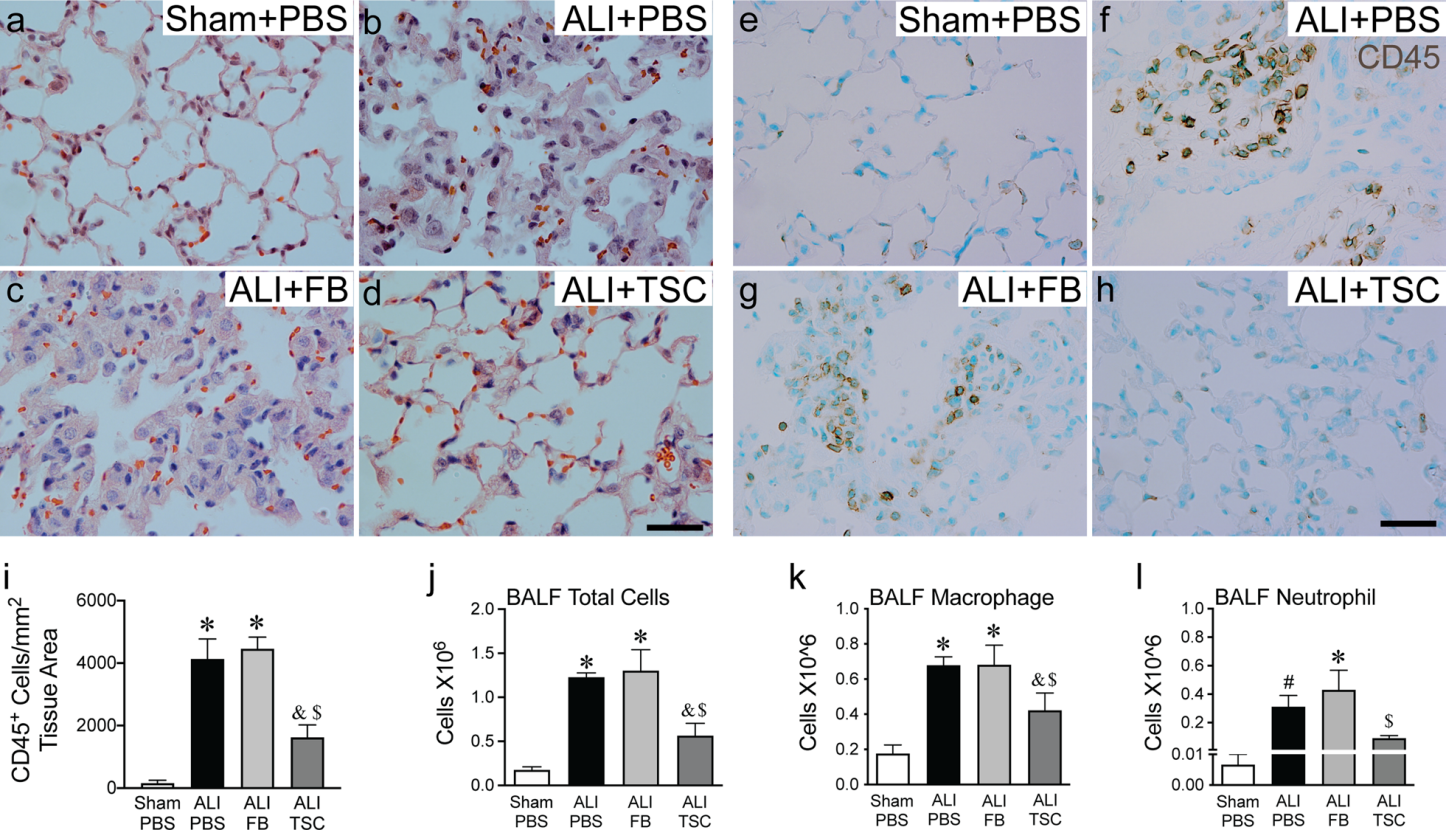
Administration of TSCs during ALI attenuates infiltration of inflammatory cells. Male C57BL/6 mice received PBS (sham) or BLM (ALI) intratracheally. At day 3 after exposure, sham mice were administrated intratracheally with PBS (sham + PBS), and ALI mice with PBS (ALI + PBS), FBs (ALI + FB) or TSCs (ALI + TSC). Lung tissue and BALFs were collected at day 7. **a**–**d** Representative images of H&E staining of the lungs in each group. **e**–**h** Representative images of immunostaining for CD45 (brown) in each group, and the lung sections were counterstained with methyl green (blue). Scale bars represent 50 µm. **i** Quantitation of CD45 immunostaining. **j**–**l** Quantification of total cell number (**j**, *n* = 5 ~ 6 for each group), macrophages (**k**, *n* = 3 ~ 6 for each group) and neutrophils (**l**, *n* = 3 ~ 6 for each group) in BALF, with the data presented as mean ± SEM. One-way analysis of variance was performed for **i**–**l**. *P < 0.05 versus sham group, ^&^*P* < 0.05 versus ALI + PBS group, ^$^*P* < 0.05 versus ALI + FB group, ^#^*P* = 0.05 versus sham group

Intratracheal BLM in mice causes ALI within the first 7 days of exposure [25, 26]. We hypothesized that transplantation of TSCs would have a benefit to decrease the injurious effect of BLM. To address this, the lungs were harvested for histology and BALF at day 7 after exposure to BLM. H&E staining was performed on the lung sections for assessment of lung injury. The lungs of mice exposed to BLM and then receiving PBS or FBs showed more inflammatory cell infiltration and more alveolar thickening, compared with sham mice receiving PBS. In contrast, the lungs of ALI mice that received TSCs revealed much less cellularity compared with mice receiving PBS or FBs, and the alveolar appearance was similar to sham mice (Fig. 1a–d). Additionally, immunostaining for CD45, a marker of inflammatory cells, confirmed that intratracheal exposure to BLM resulted in an increase in CD45 positive cells infiltrating into the lungs, compared with sham, while there were significantly less CD45 positive cells in the lungs of ALI mice that received TSCs compared with ALI mice receiving PBS or FBs (Fig. 1e–i).

We next examined total white cell counts and types of innate immune cells in BALF. BLM exposure increased the total cell counts in BALF from ALI mice receiving PBS or FBs compared with the sham. Intratracheal transplantation of TSCs significantly decreased the total cell counts in BALF, compared with ALI mice that received PBS or FBs (Fig. 1j). Infiltration of neutrophils and macrophages into the acutely injured lungs of mice after intratracheal exposure to BLM is well-described [27, 28]. Thus, BAL cells were assessed using cytospins and Wright-Giemsa staining*.* The total macrophage counts in BALF from ALI mice that received PBS or FBs were significantly increased compared with sham, while ALI mice that received TSCs had a decrease in macrophage counts compared with ALI mice receiving PBS or FBs (Fig. 1k). The total neutrophil counts in BALF from ALI mice treated with TSCs were comparable to sham, and significantly less than that of ALI mice that received FBs (Fig. 1l). Taken together, these data demonstrate that intratracheal transplantation of TSCs reduced the inflammatory response, including the reduction in neutrophils and macrophages infiltrating into the acutely injured lungs of mice exposed to BLM.

### Transplantation of TSCs attenuates pro-inflammatory cytokines/chemokines in ALI

**Fig. 2:**
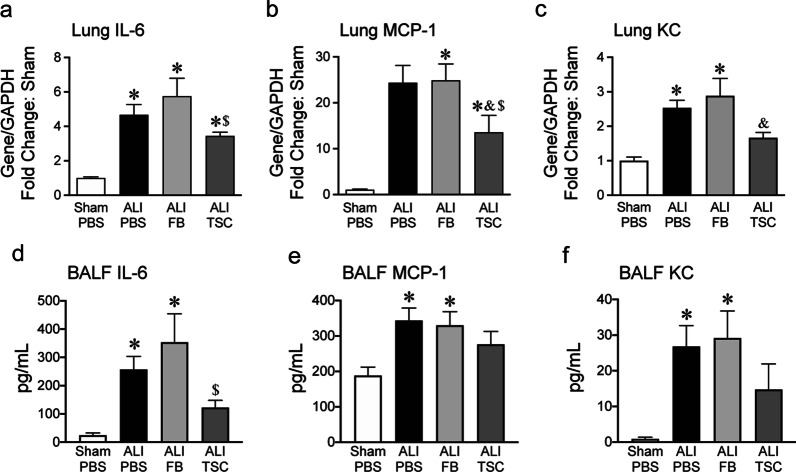
Administration of TSCs alters the cytokine profile in ALI. The lungs and BALF, from sham mice (white bars, sham + PBS), ALI mice that received PBS (black bars, ALI + PBS), FBs (light grey bars, ALI + FB) or TSCs (dark grey bars, ALI + TSC), were harvested at day 7. Quantification of qRT-PCR for **a** IL-6, **b** MCP-1 and **c** KC of the lungs was performed, and the data are presented as mean ± SEM. One-way analysis of variance was performed. *P < 0.001 versus sham group, ^&^*P* < 0.05 versus ALI + PBS group, ^$^*P* < 0.005 versus ALI + FB group, with *n* = 6 ~ 9 for each group. Quantitation of Luminex for **d** IL-6, **e** MCP-1 and **f** KC in BALF was performed, and the data are presented as mean ± SEM. One-way analysis of variance was performed. ^&^*P* < 0.05 versus ALI + PBS group, ^$^*P* < 0.05 versus ALI + FB group, *n* = 4–5 for each group

In response to BLM-induced ALI, neutrophils, macrophages, alveolar epithelial cells and endothelial cells secrete pro-inflammatory cytokines and chemokines [29, 30]. Thus, we additionally investigated whether TSCs mediated the inflammatory response to ALI induced by BLM exposure. Total RNA was isolated from lungs and qRT-PCR was performed for IL-6, MCP-1 and KC. IL-6 is a marker of inflammation in the injury phase of ALI [31, 32]. Intratracheal exposure to BLM significantly increased IL-6 expression in the lungs of ALI mice receiving PBS and FBs compared with sham lungs. Interestingly, the level of IL-6 expression was decreased in the lungs of ALI mice receiving TSCs, compared with ALI mice receiving FBs (Fig. 2a). MCP-1, one of the key chemokines that regulate migration and infiltration of monocytes/macrophages [30] was increased in ALI, while administration of TSCs significantly decreased MCP-1 expression compared with lungs of ALI mice that received PBS or FBs (Fig. 2b). KC, a factor contributing to neutrophil recruitment to the lungs [29], also was increased in the lungs of mice exposed to BLM, and treatment with TSCs after ALI resulted in a decreased level of KC compared with lungs of mice receiving FBs (Fig. 2c). Furthermore, BALF was collected from sham and ALI mice, and assessed by Luminex assay. BALF IL-6 was increased in mice exposed to BLM that received PBS or FBs, compared to sham. Intratracheal transplantation of TSCs reduced the level of BALF IL-6 compared with mice that received FB after ALI, and a trend for a decrease compared with the PBS group (Fig. 2d). MCP-1 in BALF was also increased in ALI mice receiving PBS and FBs, compared to sham. The intratracheal transplantation of TSCs had a trend for a decrease the level of BALF MCP-1, compared with mice that received PBS and FBs after BLM (Fig. 2e). In addition, KC in BALF was elevated in ALI mice receiving PBS and FBs, compared to sham. There was a trend for a decrease in KC in the BALF of ALI mice receiving TSCs compared with mice that received PBS and FBs after BLM, and the level of KC was not significantly different from sham after administration of TSC (Fig. 2f). The levels of IL-1α, IL-23, macrophage inflammatory protein (MIP) γ, FMS-like tyrosine kinase 3 (FLT3) and granulocyte colony stimulating factor (G-CSF) were found to be increased in BALF of ALI mice receiving PBS or/and FBs, while intratracheal transplantation of TSCs reduced the level of IL-1α and FLT3, but not others. The level of IL-10, IL-12p40, MIP1α, MIP1β, MIP2, interferon (IFN) γ and tumor necrosis factor (TNF) α was not significantly different between the groups (Additional File 1).

To investigate the effect of TSCs on inflammatory mediators produced by lung epithelial cells after BLM-induced injury, independent of immune cells, MLE-12 cells were exposed to BLM and treated with PBS or TSC conditioned medium (CM) for 24 h in vitro. qRT-PCR assay demonstrated that BLM induced an increase of IL-6, MCP1 and KC expression in MLE-12 cells; however, TSC-CM treatment did not diminish the elevated levels of IL-6, MPC1 and KC induced by BLM, compared to control MLE-12 cells (Additional File 2).

### TSCs protect lung epithelial cells from apoptosis after exposure to BLM in vivo and vitro

**Fig. 3:**
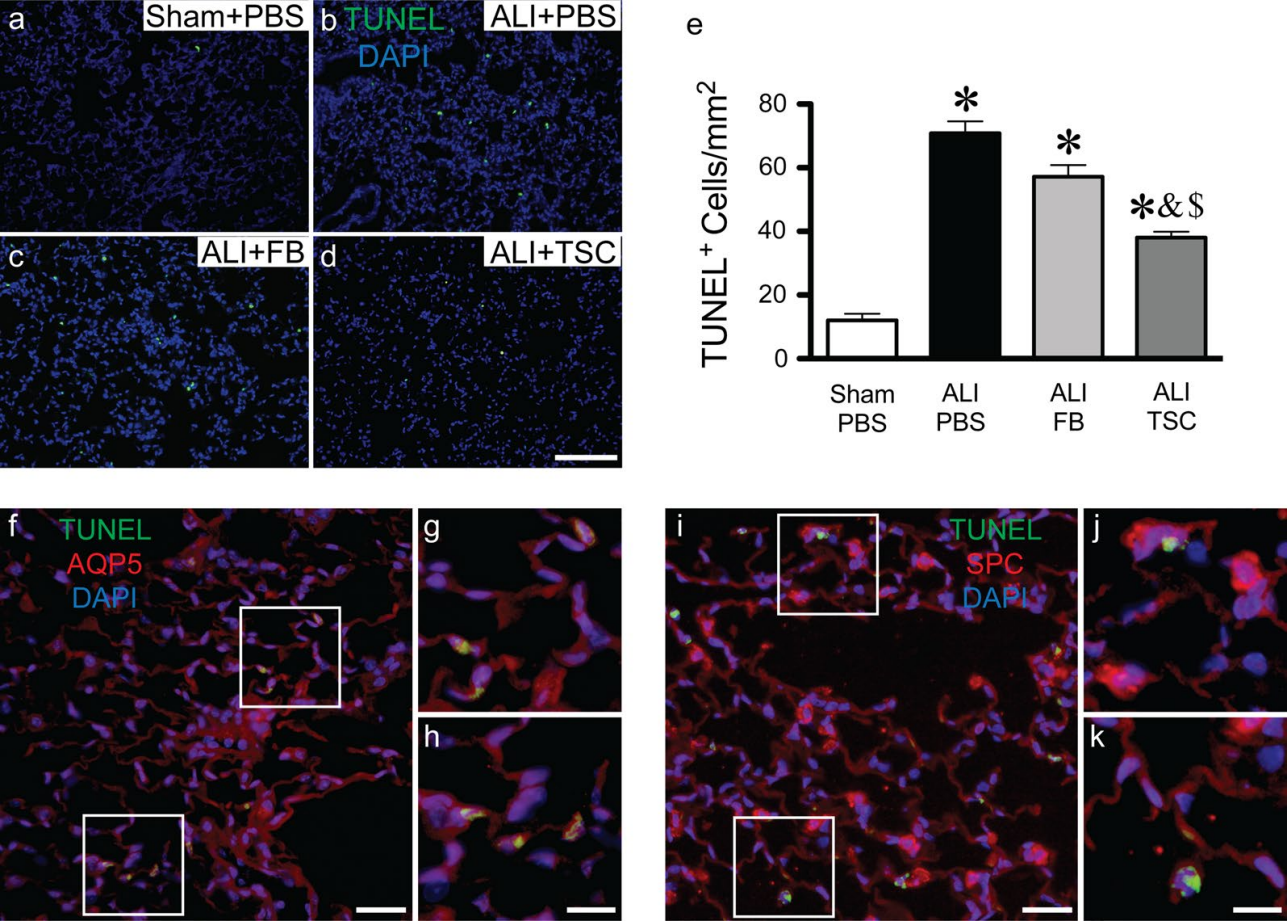
Administration of TSCs protects from apoptosis in ALI. The lungs from sham mice (**a**, sham + PBS) and ALI mice that received PBS (**b**, ALI + PBS), FBs (**c**, ALI + FB) or TSCs (**d**, ALI + TSC) were harvested at day 7. **a**–**d** Representative images of immunofluorescence staining for TUNEL (green) and DAPI (blue). **e** Quantitation of TUNEL staining, and the data are presented as mean ± SEM. One-way analysis of variance was performed, from lungs of sham + PBS (*n* = 3); ALI + PBS (*n* = 5), ALI + FB (*n* = 6), and ALI + TSC (*n* = 5). *P < 0.0001 versus sham + PBS group, ^&^*P* < 0.0001 versus ALI + PBS group, ^$^*P* < 0.001 versus ALI + FB group. **f**–**h** Representative images of immunofluorescence co-staining for TUNEL (green) and AQP5 (red, **f**–**h**) or SPC (red, **i**–**k**), and the lung sections were counterstained with DAPI (blue), on the lung sections from ALI + PBS group. The higher magnification image **g** and **h** from the white boxes in **f**, **j** and **k** from the white boxes in **i**. Scale bars represent 50 µm

The death of alveolar epithelial cells has been observed in ALI induced by BLM [24, 33]. To assess the role of TSCs in protection of the alveolar epithelial cells from apoptosis during ALI induced by BLM, we stained the lungs for cell death, using TUNEL. Apoptotic cells were frequently observed in the lungs at 7 days after intratracheal exposure to BLM in mice that received PBS or FBs, and rarely seen in the lungs of sham mice receiving PBS. Transplantation of TSCs into the lungs of mice after exposure to BLM showed a significant decrease in apoptotic cells compared with ALI mice receiving PBS and FBs (Fig. 3a–e). These data demonstrate that TSCs play a role in protecting the lungs from cell death due to BLM. To demonstrate that lung alveolar epithelial cells account for much of this apoptotic response, lung tissues were co-stained for TUNEL along with AQP5 or SPC. We found that most of the TUNEL positive cells stained positive for AQP5 (Fig. 3f–h), or for SPC (Fig. 3i–k).

**Fig. 4:**
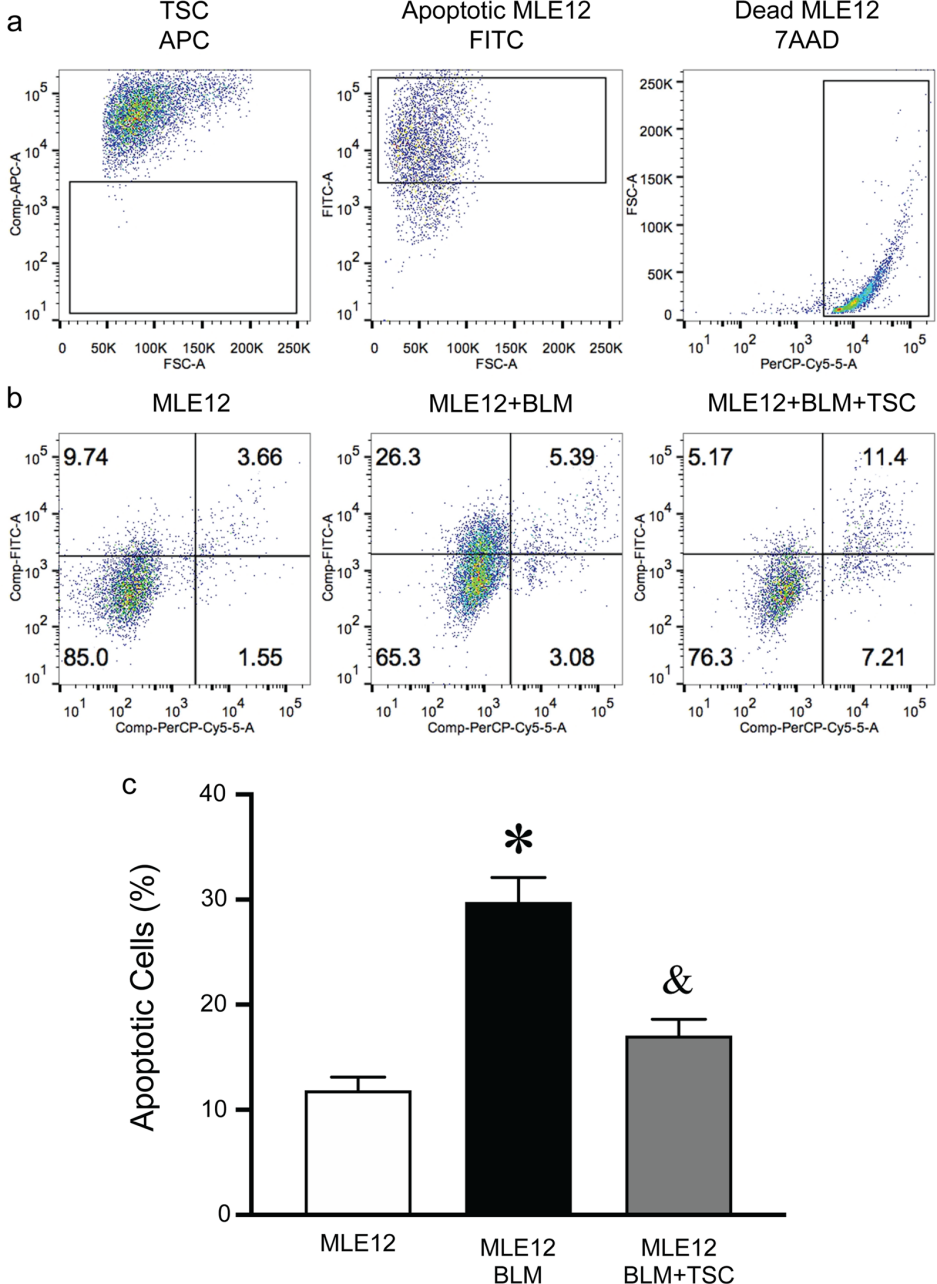
TSCs protect MLE-12 from apoptosis induced by BLM in vitro*.* MLE-12 were treated with PBS (MLE12) or BLM (MLE12 + BLM), and then co-cultured with TSCs (MLE12 + BLM + TSC). **a** The gating strategy of flow cytometry. Cells were stained for Sca1 to identify TSCs for exclusion (APC, left). MLE-12 cells were incubated at 60 °C for 1 h to induce apoptosis, and then staining for Annexin V (FITC, middle). MLE-12 cells were treated with Triton-100 for 30 min to induce cell death and staining for 7AAD (PerCP-cy5.5, right). **b** Representative scatter plots of flow cytometry in each group. **c** Quantitation of flow cytometry assay, and the data are presented as mean ± SEM. One-way analysis of variance was performed. *P < 0.0001 versus MLE12 group. ^&^*P* < 0.001 versus MLE12 + BLM group. *n* = 6 for each group

The previous data (Figs. 1 and 2) demonstrated that intratracheal transplantation of TSCs reduced inflammatory cell infiltrates and the level of pro-inflammatory cytokines, which may contribute to a decrease in parenchymal lung cell death. To understand whether TSCs may have a direct role in protecting alveolar epithelial cells, independent of immune cells and cytokines, we performed apoptosis assays in vitro. MLE-12 cells were treated with PBS or BLM for 24 h and were then co-cultured with PBS or TSCs for 16 h. Cells were harvested and stained for Sca-1, which is present in 99% of TSCs [19] and not expressed in MLE-12 cells, to exclude TSCs in the flow cytometry analysis for apoptosis. MLE-12 cells were gated to assay for apoptotic cells (Fig. 4a). BLM exposure significantly increased the apoptosis of MLE-12 cells to 29.77 ± 2.32%, compared with 11.85 ± 1.28% at baseline, while BLM-injured MLE-12 cells co-cultured with TSCs, demonstrated a significant decrease in apoptotic cell counts to 17.06 ± 1.56% (Fig. 4b, c). These data suggest TSCs directly protect lung epithelial cells from death induced by BLM.

The damage of lung epithelial cells and the infiltration of inflammatory cells into the lung subsequently result in pulmonary fibrosis, which begins to appear by 14 days after exposure to BLM [21]. Therefore, we further evaluated the effect of TSCs on lung fibrosis at 14 days after exposure to BLM. Masson’s Trichrome staining of the ALI lungs that received TSCs revealed significantly decreased lung fibrosis than that of the ALI lungs that received PBS or FBs, and comparable to sham group (Additional File 3).

### Transplanted TSCs engraft and differentiate into alveolar epithelial cells during ALI in vivo

Beyond mediating the inflammatory and injury response associated with ALI due to BLM, we next wanted to understand whether TSCs are capable of reparation of the lung epithelia by engrafting and differentiating during ALI. We first investigated in vitro the property of self-renewal and differentiation of TSCs into lung alveolar epithelial cells. TSCs formed clones from single cells (Additional File 4a) and the majority of clonal cells continued to express CD117 (Additional File 4b), demonstrating that the TSCs possess the stem cell properties of clonogenesis and self-renewal. TSCs were next cultured in differentiation medium on an air–liquid interphase and expressed AQP5 (Additional File 4c, d) or SPC (Additional File 4e, f). Total RNA was isolated from these cells and qRT-PCR revealed ~ fourfold and ~ fivefold greater mRNA levels of AQP5 and SPC, respectively, in the differentiated versus the undifferentiated cells (Additional File 4 g, h). These data demonstrate that TSCs possess the ability to differentiate into lung alveolar epithelial type I and II cells in vitro*.*

**Fig. 5:**
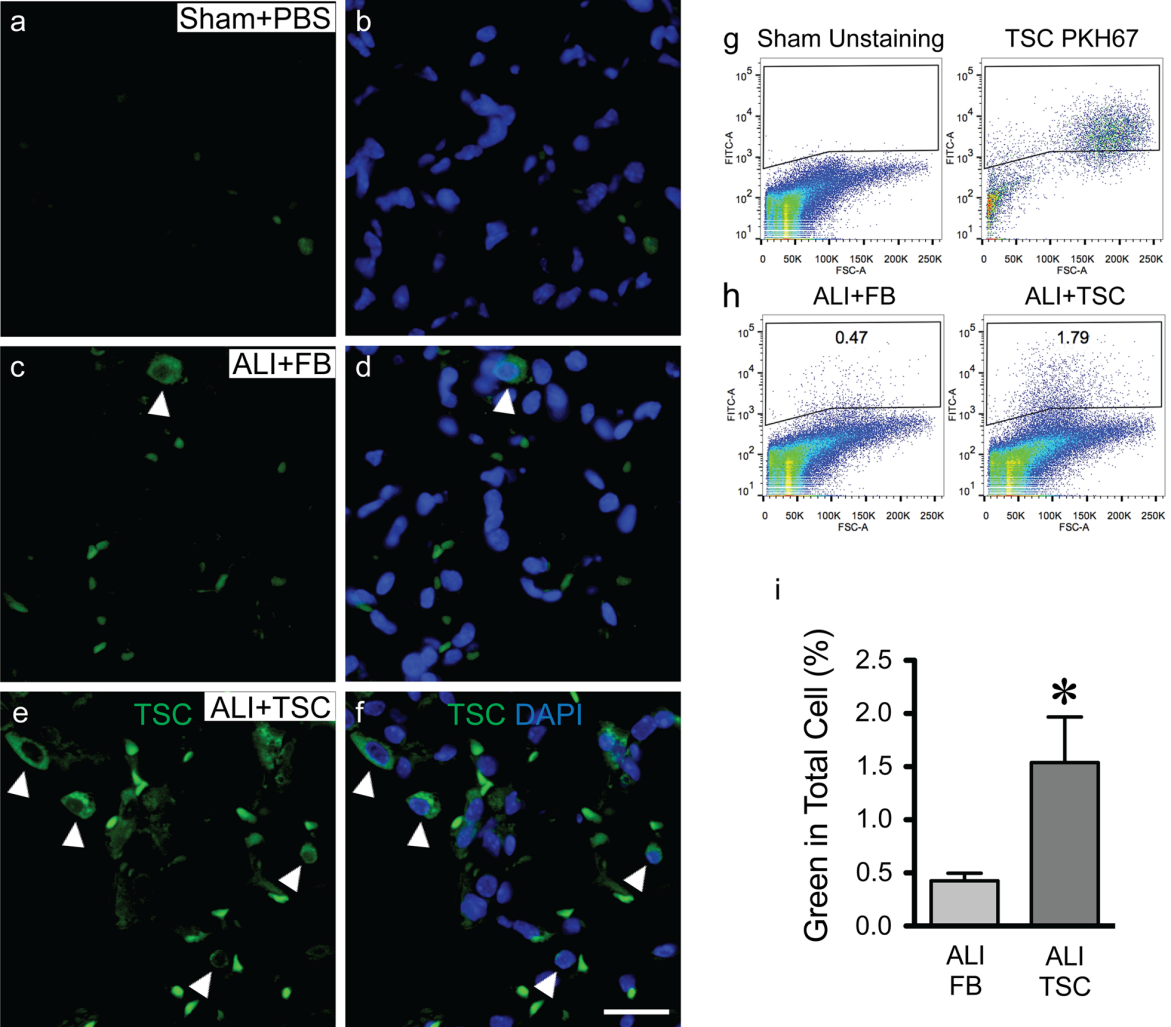
TSCs engraft in the lung injured by BLM. The lungs were harvested at day 7 after BLM exposure. **a**–**f** Representative images of the lung, from sham mice (sham + PBS, **a** and **b**), ALI mice that received FBs (ALI + FB, **c** and **d**) and TSCs (ALI + TSC, **e** and **f**). Images showing cells in FITC channel (**a**, **c** and **e**), and merged with nuclear staining for DAPI (blue, **b**, **d** and **f**). White arrowheads point to green cells. **g** Representative scatter plots of flow cytometry showing the gating strategy: FITC negative (left) and PKH67 positive (right). **h** FITC^+^ cells from the lungs of ALI mice that received FBs (ALI + FB, left) and TSCs (ALI + TSC, right). **i** Bar graph represents quantitation of % green cells in the total lung suspension, and the data is presented as mean ± SEM. Student’s unpaired *t* test was performed. *P < 0.05, versus ALI + FB group. *n* = 6 for ALI + FB and *n* = 5 for ALI + TSC

To investigate this further in vivo, we traced transplanted TSCs or FBs, which were pre-labeled with PKH67 fluorescence dye (green). Before transplantation of the cells, we investigated whether these dyes could leak out of one cell and be taken up by a neighboring cell. We co-cultured TSCs and MLE-12 cells, pre-labeled with PKH67 (green) and PKH26 (red), respectively, for 3 days. Imaging of the cells demonstrated no overlap of and green and red fluorescent dyes (Additional File 5), suggesting no transfer of dyes between cells. Next, mice received PBS (sham) or BLM (ALI) intratracheally. At day 3 after exposure to BLM, pre-labeled TSCs or FBs (green), were intratracheally administrated to ALI mice. The mice were sacrificed, and the lungs were harvested at day 7 after exposure to BLM, for histologic assessment. Mouse lungs were sectioned (5 μm) and images were taken by fluorescent microscopy. No FITC positive cells were observed in sham mice receiving PBS (Fig. 5a, b), and few cells were present in the lungs of ALI mice receiving FBs (Fig. 5c, d). However, FITC positive cells were observed in the distal lung of mice receiving BLM and subsequently transplanted with TSCs (Fig. 5e, f). Single cell suspensions of lung tissue were also harvested to quantify exogenous pre-labeled FBs and TSCs by flow cytometry. 1.54 ± 0.9% of total lung cells were found to be exogenous TSCs (green cells) in the lungs of ALI mice (day 4 after TSC administration), which was significantly greater compared with that of ALI mice that received FBs with 0.43 ± 0.2% green cells (Fig. 5g–i).

**Fig. 6:**
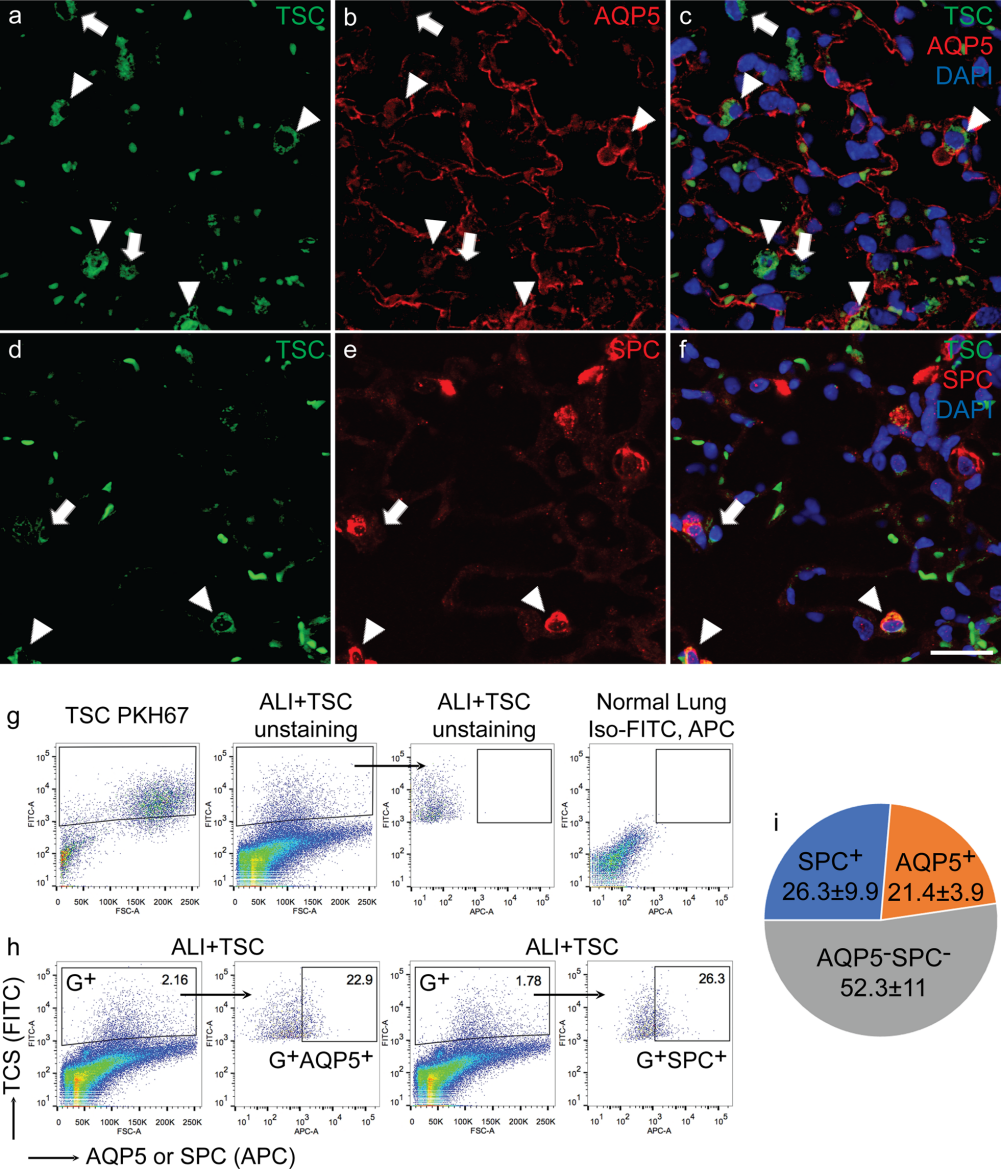
TSCs differentiate into alveolar epithelial cells in the lung injured by BLM. The lungs were harvested at day 7 after BLM exposure. **a**–**f** Representative images of the lung receiving TSC pre-labeled with PKH67 (green, **a** and **d**), and immunofluorescence staining for AQP5 and SPC (red, **b** and **e**), and merged images with nuclear staining for DAPI (blue, **c** and **f**). White arrowheads point to green^+^AQP5^+^ and green^+^SPC^+^ cells (**a**–**c** and **d**–**f**, respectively). White arrows point to green^+^AQP5^–^and green^+^SPC^–^ cells (**a**–**c** and **d**–**f**, respectively). Scale bar represents 25 µm. **g** Representative scatter plots of flow cytometry showing the gating strategy, FITC^+^ control from TSCs stained with PKH67 (first panel), FITC^+^ TSCs gated from unstained cells of the lung that received PKH67-pre-labeled TSCs (second panel), APC^+^ gated from the population of FITC^+^ cells in the second panel (third panel), and isotype control from the normal lung stained with FITC- and APC-isotypes (fourth panel). **h** Representative scatter plots of flow cytometry showing that the cells from BLM injured lungs receiving pre-labeled TSCs (FITC) were stained for AQP5 or SPC (APC). The percentage of FITC^+^ cells in the total lung cell population is shown in the gates (G+, the first and third panels), in which these cells gated for a percentage of FITC^+^AQP5^+^ and FITC^+^SPC^+^ in total FITC^+^ cells (second and fourth panels, respectively). **i** The pie graph shows quantitation of FITC^+^AQP5^+^ and FITC^+^SPC^+^ in the total FITC^+^ cells. *n* = 5 for each group

To further understand the fate of transplanted TSCs in vivo, the lung sections were stained for AQP5 and SPC. Images showed that a population green fluorescent cells expressed either AQP5 (white arrowheads, Fig. 6a–c) or SPC (white arrowheads, Fig. 6d–f), while others remained positive for green fluorescent dye and did not express AQP5 or SPC (white arrows, Fig. 6a–f). For further quantitation of the subpopulations of exogenous green cells, the single cell suspensions from the lungs were stained for AQP5 or SPC and then run for flow cytometry. In the total population of green cells, 21.38 ± 3.9% and 26.32 ± 9.9% of cells expressed AQP5 and SPC, respectively (Fig. 6g–i). These data revealed that TSCs were able to engraft and differentiate into alveolar epithelial cells during ALI.

## Discussion

The use of stem cells as a potential treatment strategy for ALI is an evolving field of investigation. MSCs from various sources and placental amnion epithelial cells have been shown to have therapeutic potential in rodent lung injury models, although there is little evidence of these exogenous stem cells populating the lung alveolar epithelium in vivo [7, 34–36]. Allogeneic MSCs are presently being used in numerous clinical trials, and while there is evidence that these cells may be immune evasive, they are not entirely immune privileged, which may limit their persistence or ability to engraft in vivo [37]. An ongoing challenge to improve the utility of MSCs therapeutically is that they are inherently a heterogeneous population of cells whose characteristic may vary depending on the donor, tissue of origin, and cell preparations [38]. In present study, we isolated TSCs using CD117, a surface marker of stem cells that is expressed in both mice and humans. We previously further characterized the TSCs and demonstrated that these cells were negative for both markers of MSCs and hematopoietic stem cells [19]. Using this approach, a CD117^+^ subpopulation of trophoblast cells allows a more homogeneous collection of cells to be isolated. In addition, CD117^+^ TSCs lack expression of both MHC class I and II proteins [19], which is consistent with the cells being immune privileged, and thus less of a concern for transplant rejection of allogeneic TSCs.

The mechanism of BLM-induced ALI is not fully understood, but numerous studies have shown that the exposure of lungs to BLM may directly trigger a series of oxidative reactions leading to cell death [27, 28]. Alveolar epithelial cell injury and death are further aggravated by the infiltration of inflammatory cells into the lung [27–29, 39]. As a treatment for ALI, it is important to reduce the infiltration of inflammatory and protect alveolar epithelial cells from death. In the present study, mice receiving TSCs intratracheally demonstrated a significant decrease in CD45^+^ cells in the lung, and a reduction in total cell count, macrophages and neutrophils in BALF. Pro-inflammatory cytokines are released from various cells, such as the epithelial and endothelial cells, as well as macrophages and neutrophils [29–32]. IL-6 is considered to be an important pro-inflammatory cytokine and a biomarker of poor outcome in ALI [31, 32]. MCP-1 is one of the key chemokines that regulates migration and infiltration of monocytes/macrophages [30], and KC is one of the major chemokines contributing to neutrophil recruitment to the lung [29]. Our data demonstrated a decrease in the expression of IL-6, MCP-1 and KC in the lungs, and reduced levels of these mediators (particularly IL-6) in BALF from ALI mice that received TSCs. Screening for a panel of inflammatory mediators in BALF also demonstrated a decrease in IL-1α and FLT3, known mediators of the pathobiology of ALI [40–42], after TSC administration. This study demonstrates for the first time that TSCs are capable of decreasing the expression and production of inflammatory chemokines and cytokines in BLM-induced ALI, resulting in a reduced infiltration of macrophages and neutrophils into the lungs. These data support TSCs anti-inflammatory properties in vivo. Interestingly, TSCs demonstrated no effects on the levels of IL-6, MCP1 and KC in MLE-12 cells exposed to BLM in vitro*,* suggesting that the decrease of these mediators in the lungs of ALI mice by TSCs was likely related to a decrease in immune cells infiltrating into the lungs and not BLM-induced production in lung epithelial cells. Although the TSC-derived factors promoting this anti-inflammatory response are still under further investigation, it is known that trophoblast cells derived from TSCs have been shown to produce vasoactive intestinal peptide, which modifies monocyte and macrophage phenotypes favoring a predominantly anti-inflammatory and M2-like alternatively activated profile during normal pregnancies [43]. Further investigation of macrophage polarization by TSCs in BLM-injured lung will be a worthy area for future study.

Intratracheal transplantation of TSCs into mice with BLM-induced ALI reduced the death of parenchymal lung cells, compared with mice exposed to BLM receiving PBS or FBs. This likely occurred in part by decreasing the inflammatory response in the lungs, by reduced infiltration of macrophages and neutrophils into the lungs, and the decreased production of cytokines and chemokines. However, beyond the inflammatory response leading to lung injury, our in vitro studies with MLE-12 cells exposed to BLM suggest that TSCs may also play a direct role in protecting the alveolar epithelial cells from injury and cell death.

Studies have reported the ability of murine ESCs and iPSCs to engraft and differentiate into alveolar epithelial cells in vitro and in vivo [9–11]. However, immunogenicity, tumorigenesis and ethical issues of these cells represent barriers to their clinical application. The current study demonstrates, beyond the impact of TSCs on inflammation and death of lung alveolar epithelial cells, CD117^+^ TSCs were able to differentiate into alveolar epithelial cells in vitro and in vivo [19]. At 3 days after exposure to BLM, TSCs were intratracheally transplanted into mice, and assessment of lung engraftment was performed 4 days later. Approximately 1.5% of lung cells were derived from exogenous TSCs. Some of these exogenous cells stained positive for either AQP5 or SPC, markers of alveolar epithelial type I or type II cells, respectively. In the population of exogenously labeled TSCs that engrafted, 21% expressed AQP5 and 26% expressed SPC. These results suggest that TSCs were able to survive after intratracheal transplantation into the injured lungs, and nearly half of the surviving cells differentiated into alveolar epithelial type I and II cells. Whether the engraftment of TSCs in the lungs after BLM exposure contributed to the reduction of injury requires further investigation, however this study demonstrates that TSCs have the potential to engraft during ALI. To our knowledge, no investigation of administering TSCs from term placentas during experimental ALI has been reported. Transplantation of TSCs into the hearts of mice after myocardial infarction has been showed an improvement cardiac function, comparable to that of administering MSCs, but only the TSCs exhibited the potential to differentiate into cardiomyocytes in vivo [20]. However, these TSCs were harvested at the early stage of pregnancy from the trophectoderm of blastocysts, which raises an ethical concern and limited resources compared to our harvest of TSCs from the term placenta.

The present study provides novel insight into the therapeutic use of TSCs in an experimental model of acute lung injury, utilizing exposure to a drug known to have lung toxicity. These studies are limited to animal models of disease, however the knowledge gained in preclinical models of disease may allow future studies in humans. An advantage of using CD117 to isolate TSCs is that CD117 is a marker expressed on the surface of both mouse and human cells [19], thus providing feasibility for translation to humans. In addition, exposure to BLM is just one model of ALI that also leads to lung fibrosis. While our studies using TSCs demonstrate a decrease in ALI, future studies will need to be performed to further understand the effect of TSCs on the later consequences of BLM to promote lung fibrosis. It will also be important to study other experimental models of acute lung injury, including models involving pathogens or products of bacteria such as lipopolysaccharide. Clearly, the pathophysiologic consequences and therapeutic approach to a toxic chemical or drug exposure may be different than resolving an infectious insult.

## Conclusions

Taken together, our data provide preclinical evidence that TSCs may have future clinical application in ALI by decreasing the inflammatory response, reducing the death of lung alveolar epithelial cells, and engrafting with the potential for differentiation and replacement of injured cells in the lung.

## Supplementary Information

**Additional file 1.** Administration of TSCs alters the cytokine profile of BALF in ALI. BALF, from sham mice (white bars, sham+PBS), ALI mice that received PBS (black bars, ALI+PBS), FBs (light grey bars, ALI+FB) or TSCs (dark grey bars, ALI+TSC), were harvested at day 7. Undiluted sample of 20 μL was used for analysis by Luminex FlexMap 3D assay system per manufacturer’s instructions. Quantification was performed for a) IL-1α, b) IL-10, c) IL-12p40, d) IL-23, e) MIP1α, f) MIP1β, g) MIPγ, h) MIP2, i) IFNγ, j) TNFα, k) FLT3 and l) GCSF. The data are presented as mean±SEM. One-way analysis of variance was performed. *P<0.05 versus sham group, &P<0.05 versus ALI+PBS group, n = 4–5 for each group.

**Additional file 2.** TSCs-CM does not alter inflammatory mediators in MLE-12 cells exposed to BLM in vitro. MLE-12 cells were treated with PBS (MLE12), or 50ng/mL BLM with or without TSC conditioned medium (MLE12+BLM or MLE12+BLM+CM, respectively) for 24 hours. Total RNA was extracted and qRT-PCR was performed. Quantitation of qRT-PCR of IL-6 (a), MCP-1 (b) and KC (c) in MLE-12 cells received with PBS only (white bars), BLM (black bars) or BLM and TSC-CM (gray bars). The data are presented as mean±SEM. One-way analysis of variance was performed. *P<0.05 versus MLE12 group, n = 5–8 for each group.

**Additional file 3.** TSCs attenuate BLM-induced lung fibrosis. The lungs were harvested at day 14 after BLM exposure. a~d) Representative image of Masson’s trichrome blue staining for the lung. Scale bar represents 50µm. e) Quantitation of Masson’s trichrome blue staining of the lung. Following Masson’s trichrome staining, tissues were scanned using VS120 slides scanner, at the fully automated mode and 10x objective (39). Images were uploaded in ImgaeJ software. The collagen deposition was expressed as a percentage of collagen in total tissue area, and presented as mean±SEM. One-way analysis of variance was performed. *P<0.005 versus sham group. &P<0.05 versus ALI+PBS group, n = 3~4 mice for each group.

**Additional file 4.** TSC possess the capability of clone formation and differentiation of into alveolar epithelial cells in vitro. TSCs were cultured in a 100-mm dish at limited dilution of one cell every 60mm2 for 14 days. Representative phase contrast images of an entire clone (a), and a clone immunofluorescence stained for CD117 (green) and DAPI (blue, b). TSCs were seeded on the apical side of a clear12-transwell plate and exposed to differentiation medium of 2% FBS DMEM/F12 supplied with 0.005mg/mL insulin, 0.01mg/mL Tranferrin, 30nM Sodium selenite, 10nM Hydrocortisone, 10nM Beta-estradiol, 10nM HEPES, 2mM L-glutamine and 50ng/mL EGF for 6 days, following air-liquid interface for an additional 11-12 days. Representative images of these differentiated cells immuno-fluorescence stained for AQP5 (red, c and d), SPC (red, e and f). Scale bars represent 1000µm for a and b, 50µm for c~f. Bar graphs showing quantitation of qRT-PCR for AQP5 (g) and SPC (h) in fold change comparing differentiated (Diff, black bars) to undifferentiated cells (Undiff, white bars). The data are presented as mean±SEM, and analysis was performed by Student’s unpaired t test. *versus undifferentiated group, P<0.001 for AQP5, P<0.05 for SPC, n=5 for each group.

**Additional file 5.** Assessment of PKH67 dye leakage into surrounding cells in vitro. TSCs and MLE-12 cells were pre-labeled with PKH67 (green) and PKH26 (red), respectively. TSCs were mixed with MLE-12 cells at a ratio of 1:10 and co-cultured for 3 days. Cells were harvested and a cytospin performed to concentrate the cells. Representative image showing a) mixture unlabeled MLE-12 cells and TSCs staining for DAPI (blue); b) TSCs pre-labeled with PKH67; c) MLE-12 cells pre-labeled with PKH26; d~f) co-cultured pre-labeled TSCs and MLE-12 cells in FITC channel (d), in red channel (e) and merged image of green, red and blue (DAPI, f). White arrowheads point to the green TSCs and arrows point to the red MLE-12 cells. Scale bar represents 50µm.

## Data Availability

The original data are available from the corresponding author on request.

## Abbreviations

ALI: Acute lung injury
AQP5: Aquaporin 5
ARDS: Acute respiratory distress syndrome
BALF: Bronchoalveolar lavage fluid
BLM: Bleomycin
CCL: Chemokine (C–C motif) ligand
CO: Carbon monoxide
CM: Conditioned medium
DAPI: 4′,6-Diamidino-2-phenylindole
ELISA: Enzyme-linked immunosorbent assay
ESCs: Embryonic stem cells
FBs: Fibroblasts
FLT: FMS-like tyrosine kinase
GCSF: Granulocyte colony stimulating factor
H&E: Hematoxylin and Eosin
IL: Interleukin
iPSCs: Induced pluripotent stem cells
MIP: Macrophage Inflammatory Protein
MLE-12: Mouse lung epithelial cells
MSCs: Mesenchymal stem/stromal cells
MCP-1: Monocyte chemoattractant protein-1
KC: Keratinocyte-derived chemokine
qRT-PCR: Real-time polymerase chain reaction
SPC: Prosurfactant protein C
TSCs: Trophoblast stem cells
TUNEL: Terminal deoxynucleotidyl transferase dUTP nick end labeling

## References

[1] Johnson ER, Matthay MA (2010). Acute lung injury: epidemiology, pathogenesis, and treatment. J Aerosol Med Pulm Drug Deliv.

[2] Rubenfeld GD, Caldwell E, Peabody E, Weaver J, Martin DP, Neff M (2005). Incidence and outcomes of acute lung injury. N Engl J Med.

[3] Allawzi A, Elajaili H, Redente EF, Nozik-Grayck E (2019). Oxidative toxicology of bleomycin: role of the extracellular redox environment. Curr Opin Toxicol.

[4] Matute-Bello G, Frevert CW, Martin TR (2008). Animal models of acute lung injury. Am J Physiol Lung Cell Mol Physiol.

[5] Thompson BT, Chambers RC, Liu KD (2017). Acute respiratory distress syndrome. N Engl J Med.

[6] Moore BB, Hogaboam CM (2008). Murine models of pulmonary fibrosis. Am J Physiol Lung Cell Mol Physiol.

[7] Araujo IM, Abreu SC, Maron-Gutierrez T, Cruz F, Fujisaki L, Carreira H (2010). Bone marrow-derived mononuclear cell therapy in experimental pulmonary and extrapulmonary acute lung injury. Crit Care Med.

[8] Liu KD, Wilson JG, Zhuo H, Caballero L, McMillan ML, Fang X (2014). Design and implementation of the START (STem cells for ARDS Treatment) trial, a phase 1/2 trial of human mesenchymal stem/stromal cells for the treatment of moderate-severe acute respiratory distress syndrome. Ann Intensive Care.

[9] Soh BS, Zheng D, Li Yeo JS, Yang HH, Ng SY, Wong LH (2012). CD166(pos) subpopulation from differentiated human ES and iPS cells support repair of acute lung injury. Mol Ther.

[10] Wang D, Morales JE, Calame DG, Alcorn JL, Wetsel RA (2010). Transplantation of human embryonic stem cell-derived alveolar epithelial type II cells abrogates acute lung injury in mice. Mol Ther.

[11] Wilson JG, Liu KD, Zhuo H, Caballero L, McMillan M, Fang X (2015). Mesenchymal stem (stromal) cells for treatment of ARDS: a phase 1 clinical trial. Lancet Respir Med.

[12] Lee JW, Fang X, Krasnodembskaya A, Howard JP, Matthay MA (2011). Concise review: mesenchymal stem cells for acute lung injury: role of paracrine soluble factors. Stem Cells.

[13] Yin X, Liang Z, Yun Y, Pei L (2015). Intravenous transplantation of BMP2-transduced endothelial progenitor cells attenuates lipopolysaccharide-induced acute lung injury in rats. Cell Physiol Biochem.

[14] Antoniadou E, David AL (2016). Placental stem cells. Best Pract Res Clin Obstet Gynaecol.

[15] Pipino C, Shangaris P, Resca E, Zia S, Deprest J, Sebire NJ (2013). Placenta as a reservoir of stem cells: an underutilized resource?. Br Med Bull.

[16] Pogozhykh O, Prokopyuk V, Figueiredo C, Pogozhykh D (2018). Placenta and placental derivatives in regenerative therapies: experimental studies, history, and prospects. Stem Cells Int.

[17] Silini AR, Di Pietro R, Lang-Olip I, Alviano F, Banerjee A, Basile M, et al. Perinatal derivatives: where do we stand? A roadmap of the human placenta and consensus for tissue and cell nomenclature. Front Bioeng Biotechnol. 2020;8:610544. 10.3389/fbioe.2020.610544 PMC7773933 33392174

[18] Natale BV, Schweitzer C, Hughes M, Globisch MA, Kotadia R, Tremblay E (2017). Sca-1 identifies a trophoblast population with multipotent potential in the mid-gestation mouse placenta. Sci Rep.

[19] Hou M, Han J, Li G, Kwon MY, Jiang J, Emani S (2020). Multipotency of mouse trophoblast stem cells. Stem Cell Res Ther.

[20] Li G, Chen J, Zhang X, He G, Tan W, Wu H (2017). Cardiac repair in a mouse model of acute myocardial infarction with trophoblast stem cells. Sci Rep.

[21] Gilhodes JC, Jule Y, Kreuz S, Stierstorfer B, Stiller D, Wollin L. Quantification of pulmonary fibrosis in a bleomycin mouse model using automated histological image analysis. PLoS ONE. 2017;12(1):e0170561. 10.1371/journal.pone.0170561 PMC5249201 28107543

[22] Hall SR, Tsoyi K, Ith B, Padera RF, Lederer JA, Wang Z (2013). Mesenchymal stromal cells improve survival during sepsis in the absence of heme oxygenase-1: the importance of neutrophils. Stem Cells.

[23] Ng J, Guo F, Marneth AE, Ghanta S, Kwon MY, Keegan J (2020). Augmenting emergency granulopoiesis with CpG conditioned mesenchymal stromal cells in murine neutropenic sepsis. Blood Adv.

[24] Lee VY, Schroedl C, Brunelle JK, Buccellato LJ, Akinci OI, Kaneto H (2005). Bleomycin induces alveolar epithelial cell death through JNK-dependent activation of the mitochondrial death pathway. Am J Physiol Lung Cell Mol Physiol.

[25] Rhee CK, Lee SH, Yoon HK, Kim SC, Lee SY, Kwon SS (2011). Effect of nilotinib on bleomycin-induced acute lung injury and pulmonary fibrosis in mice. Respiration.

[26] Steffen L, Ruppert C, Hoymann HG, Funke M, Ebener S, Kloth C (2017). Surfactant replacement therapy reduces acute lung injury and collapse induration-related lung remodeling in the bleomycin model. Am J Physiol Lung Cell Mol Physiol.

[27] Williamson JD, Sadofsky LR, Hart SP (2015). The pathogenesis of bleomycin-induced lung injury in animals and its applicability to human idiopathic pulmonary fibrosis. Exp Lung Res.

[28] Su X, Liu K, Xie Y, Zhang M, Wang Y, Zhao M (2019). Protective effect of a polyphenols-rich extract from Inonotus Sanghuang on bleomycin-induced acute lung injury in mice. Life Sci.

[29] Shah D, Romero F, Stafstrom W, Duong M, Summer R (2014). Extracellular ATP mediates the late phase of neutrophil recruitment to the lung in murine models of acute lung injury. Am J Physiol Lung Cell Mol Physiol.

[30] Deshmane SL, Kremlev S, Amini S, Sawaya BE (2009). Monocyte chemoattractant protein-1 (MCP-1): an overview. J Interferon Cytokine Res.

[31] Bhargava M, Wendt CH (2012). Biomarkers in acute lung injury. Transl Res.

[32] Cross LJ, Matthay MA (2011). Biomarkers in acute lung injury: insights into the pathogenesis of acute lung injury. Crit Care Clin.

[33] Olajuyin AM, Zhang X, Ji HL (2019). Alveolar type 2 progenitor cells for lung injury repair. Cell Death Discov.

[34] Hodges RJ, Lim R, Jenkin G, Wallace EM. Amnion epithelial cells as a candidate therapy for acute and chronic lung injury. Stem Cells Int. 2012;2012:709763. 10.1155/2012/709763 PMC3345254 22577395

[35] Murphy S, Lim R, Dickinson H, Acharya R, Rosli S, Jenkin G (2011). Human amnion epithelial cells prevent bleomycin-induced lung injury and preserve lung function. Cell Transplant.

[36] Yuan W, Song HY, Xiong J, Jiang WL, Kang GJ, Huang J, et al. Placentaderived mesenchymal stem cells ameliorate lipopolysaccharideinduced inflammation in RAW264.7 cells and acute lung injury in rats. Mol Med Rep. 2020. 10.3892/mmr.2020.11231 PMC7339743 32626979

[37] Ankrum JA, Ong JF, Karp JM (2014). Mesenchymal stem cells: immune evasive, not immune privileged. Nat Biotechnol.

[38] Levy O, Kuai R, Siren EMJ, Bhere D, Milton Y, Nissar N, et al. Shattering barriers toward clinically meaningful MSC therapies. Sci Adv. 2020;6(30):eaba6884. 10.1126/sciadv.aba6884 PMC7439491 32832666

[39] White DA, Kris MG, Stover DE (1987). Bronchoalveolar lavage cell populations in bleomycin lung toxicity. Thorax.

[40] Dong L, He HL, Lu XM, Yang Y, Qiu HB (2012). Modulation of FLT3 signaling targets conventional dendritic cells to attenuate acute lung injury. APMIS.

[41] Rabolli V, Badissi AA, Devosse R, Uwambayinema F, Yakoub Y, Palmai-Pallag M (2014). The alarmin IL-1alpha is a master cytokine in acute lung inflammation induced by silica micro- and nanoparticles. Part Fibre Toxicol.

[42] Suwara MI, Green NJ, Borthwick LA, Mann J, Mayer-Barber KD, Barron L (2014). IL-1alpha released from damaged epithelial cells is sufficient and essential to trigger inflammatory responses in human lung fibroblasts. Mucosal Immunol.

[43] Ramhorst R, Calo G, Paparini D, Vota D, Hauk V, Gallino L (2019). Control of the inflammatory response during pregnancy: potential role of VIP as a regulatory peptide. Ann N Y Acad Sci.

