# Supplementary figures and images for: Intratracheal transplantation of trophoblast stem cells attenuates acute lung injury in mice
Source: Stem Cell Res Ther. 2021 Aug 30;12:487. doi: 10.1186/s13287-021-02550-z (PMC8404310; doi:10.1186/s13287-021-02550-z)

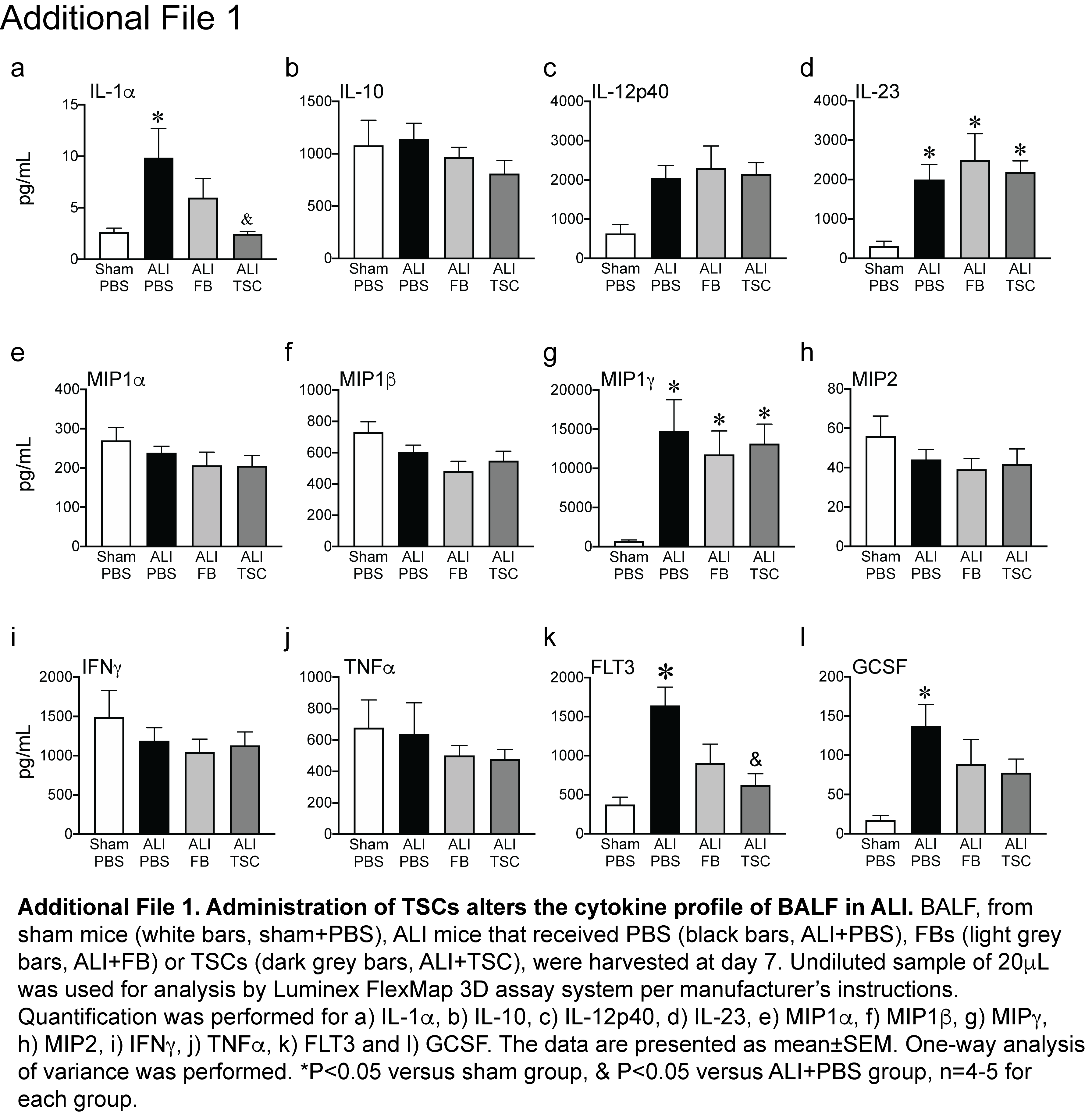

Supplement: Supplementary file 1 — Additional file 1. Administration of TSCs alters the cytokine profile of BALF in ALI. BALF, from sham mice (white bars, sham+PBS), ALI mice that received PBS (black bars, ALI+PBS), FBs (light grey bars, ALI+FB) or TSCs (dark grey bars, ALI+TSC), were harvested at day 7. Undiluted sample of 20 μL was used for analysis by Luminex FlexMap 3D assay system per manufacturer’s instructions. Quantification was performed for a) IL-1α, b) IL-10, c) IL-12p40, d) IL-23, e) MIP1α, f) MIP1β, g) MIPγ, h) MIP2, i) IFNγ, j) TNFα, k) FLT3 and l) GCSF. The data are presented as mean±SEM. One-way analysis of variance was performed. *P<0.05 versus sham group, &P<0.05 versus ALI+PBS group, n = 4–5 for each group. [file 13287_2021_2550_MOESM1_ESM.png]

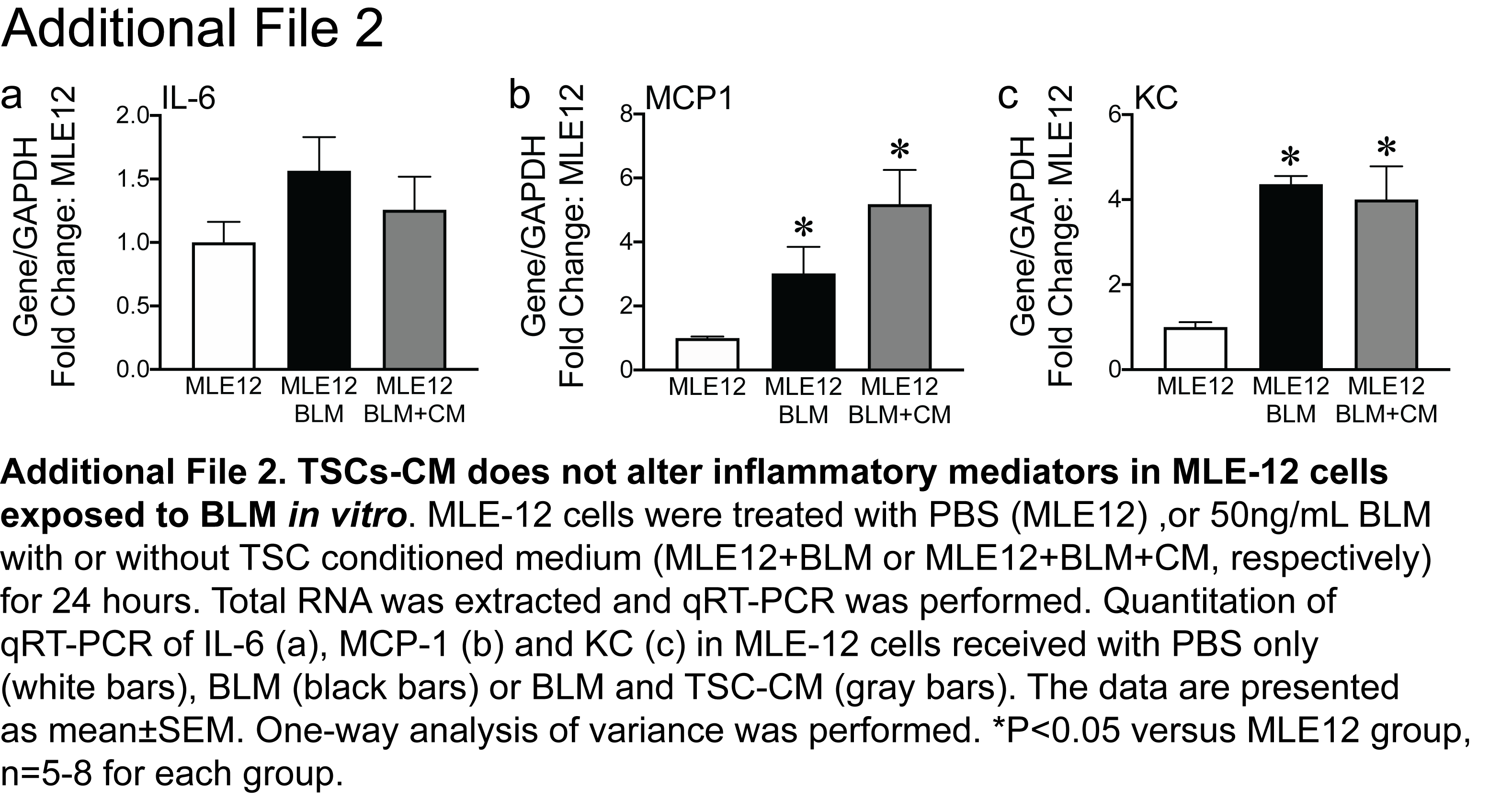

Supplement: Supplementary file 2 — Additional file 2. TSCs-CM does not alter inflammatory mediators in MLE-12 cells exposed to BLM in vitro. MLE-12 cells were treated with PBS (MLE12), or 50ng/mL BLM with or without TSC conditioned medium (MLE12+BLM or MLE12+BLM+CM, respectively) for 24 hours. Total RNA was extracted and qRT-PCR was performed. Quantitation of qRT-PCR of IL-6 (a), MCP-1 (b) and KC (c) in MLE-12 cells received with PBS only (white bars), BLM (black bars) or BLM and TSC-CM (gray bars). The data are presented as mean±SEM. One-way analysis of variance was performed. *P<0.05 versus MLE12 group, n = 5–8 for each group. [file 13287_2021_2550_MOESM2_ESM.png]

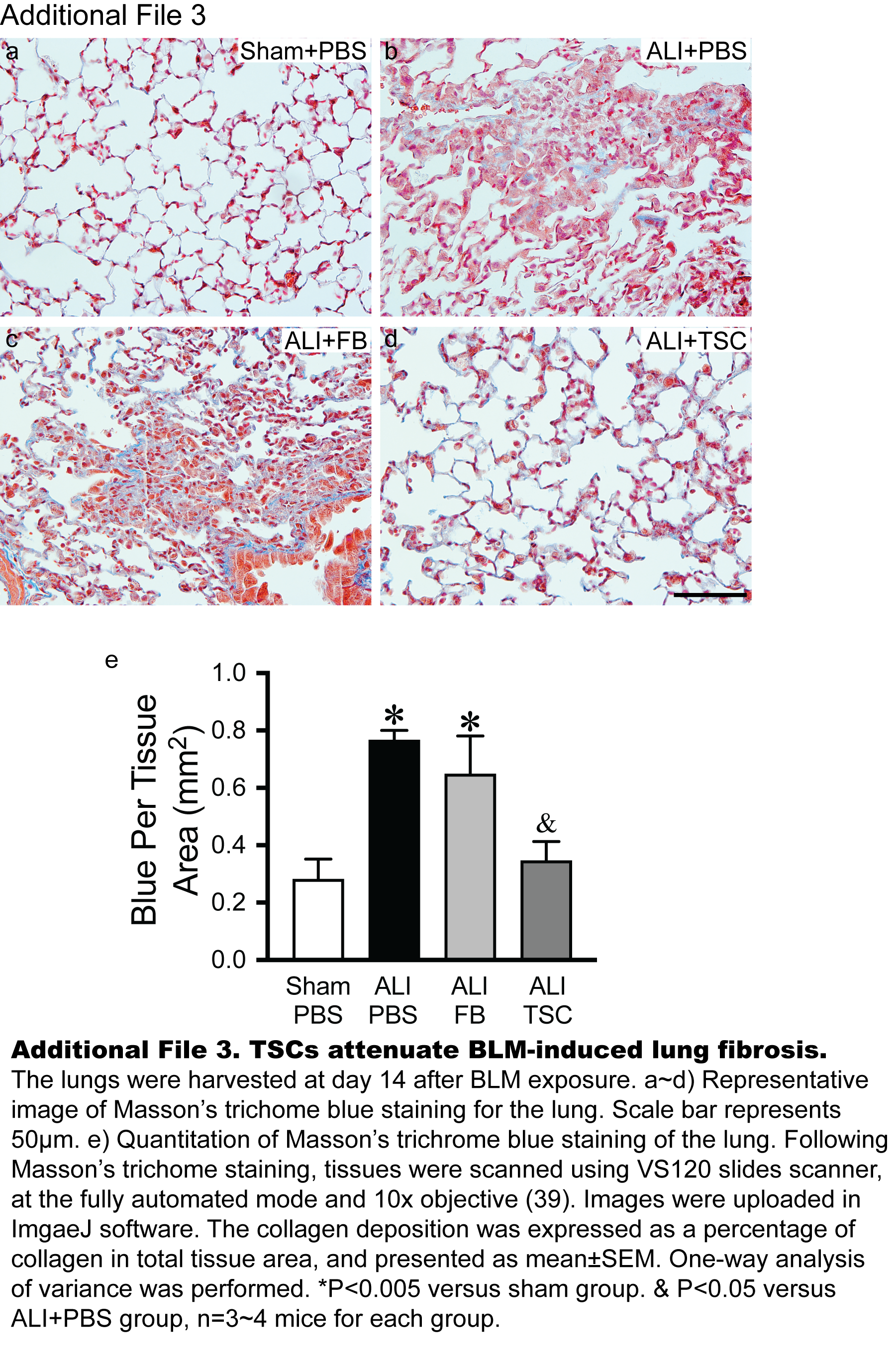

Supplement: Supplementary file 3 — Additional file 3. TSCs attenuate BLM-induced lung fibrosis. The lungs were harvested at day 14 after BLM exposure. a~d) Representative image of Masson’s trichrome blue staining for the lung. Scale bar represents 50µm. e) Quantitation of Masson’s trichrome blue staining of the lung. Following Masson’s trichrome staining, tissues were scanned using VS120 slides scanner, at the fully automated mode and 10x objective (39). Images were uploaded in ImgaeJ software. The collagen deposition was expressed as a percentage of collagen in total tissue area, and presented as mean±SEM. One-way analysis of variance was performed. *P<0.005 versus sham group. &P<0.05 versus ALI+PBS group, n = 3~4 mice for each group. [file 13287_2021_2550_MOESM3_ESM.png]

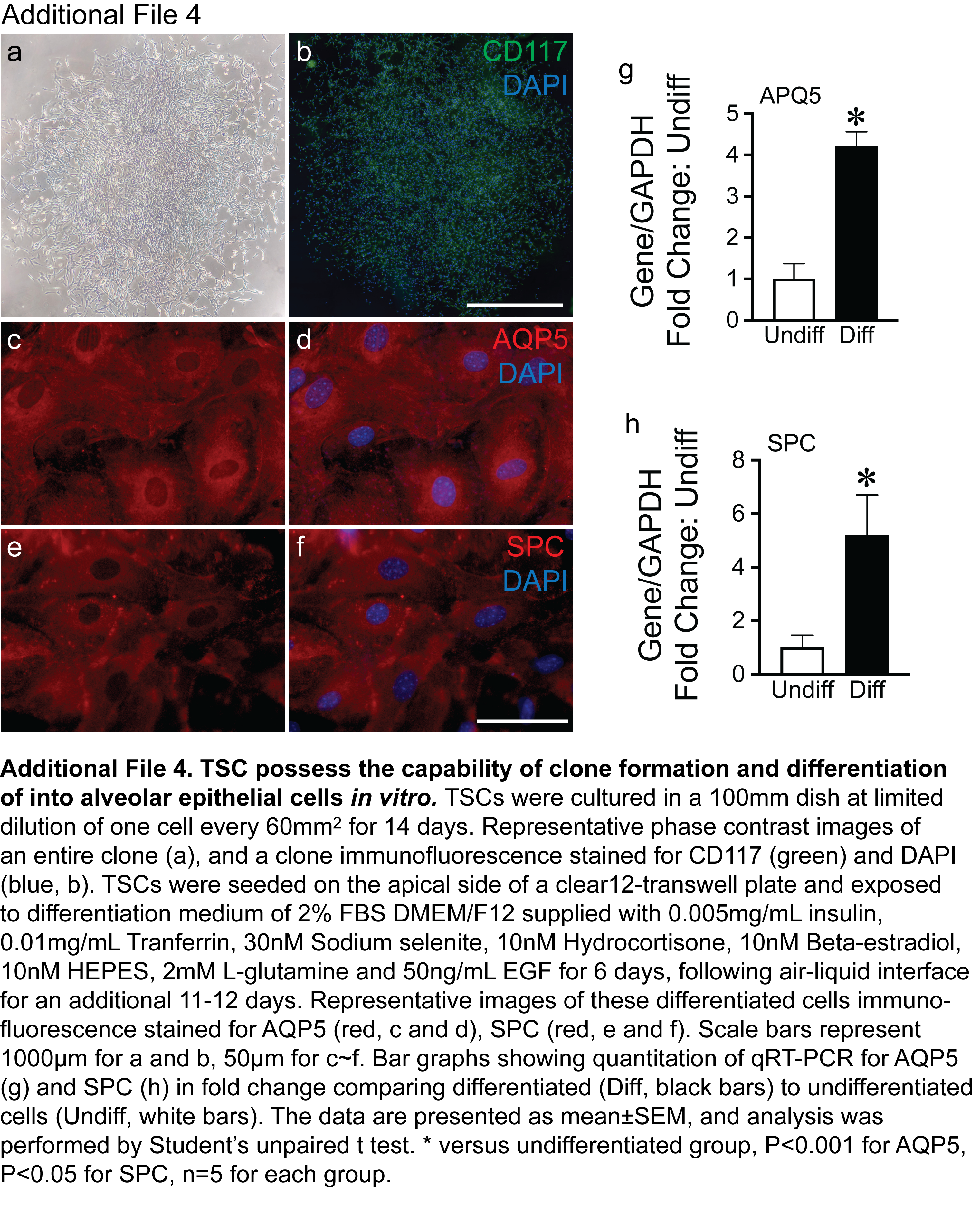

Supplement: Supplementary file 4 — Additional file 4. TSC possess the capability of clone formation and differentiation of into alveolar epithelial cells in vitro. TSCs were cultured in a 100-mm dish at limited dilution of one cell every 60mm2 for 14 days. Representative phase contrast images of an entire clone (a), and a clone immunofluorescence stained for CD117 (green) and DAPI (blue, b). TSCs were seeded on the apical side of a clear12-transwell plate and exposed to differentiation medium of 2% FBS DMEM/F12 supplied with 0.005mg/mL insulin, 0.01mg/mL Tranferrin, 30nM Sodium selenite, 10nM Hydrocortisone, 10nM Beta-estradiol, 10nM HEPES, 2mM L-glutamine and 50ng/mL EGF for 6 days, following air-liquid interface for an additional 11-12 days. Representative images of these differentiated cells immuno-fluorescence stained for AQP5 (red, c and d), SPC (red, e and f). Scale bars represent 1000µm for a and b, 50µm for c~f. Bar graphs showing quantitation of qRT-PCR for AQP5 (g) and SPC (h) in fold change comparing differentiated (Diff, black bars) to undifferentiated cells (Undiff, white bars). The data are presented as mean±SEM, and analysis was performed by Student’s unpaired t test. *versus undifferentiated group, P<0.001 for AQP5, P<0.05 for SPC, n=5 for each group. [file 13287_2021_2550_MOESM4_ESM.png]

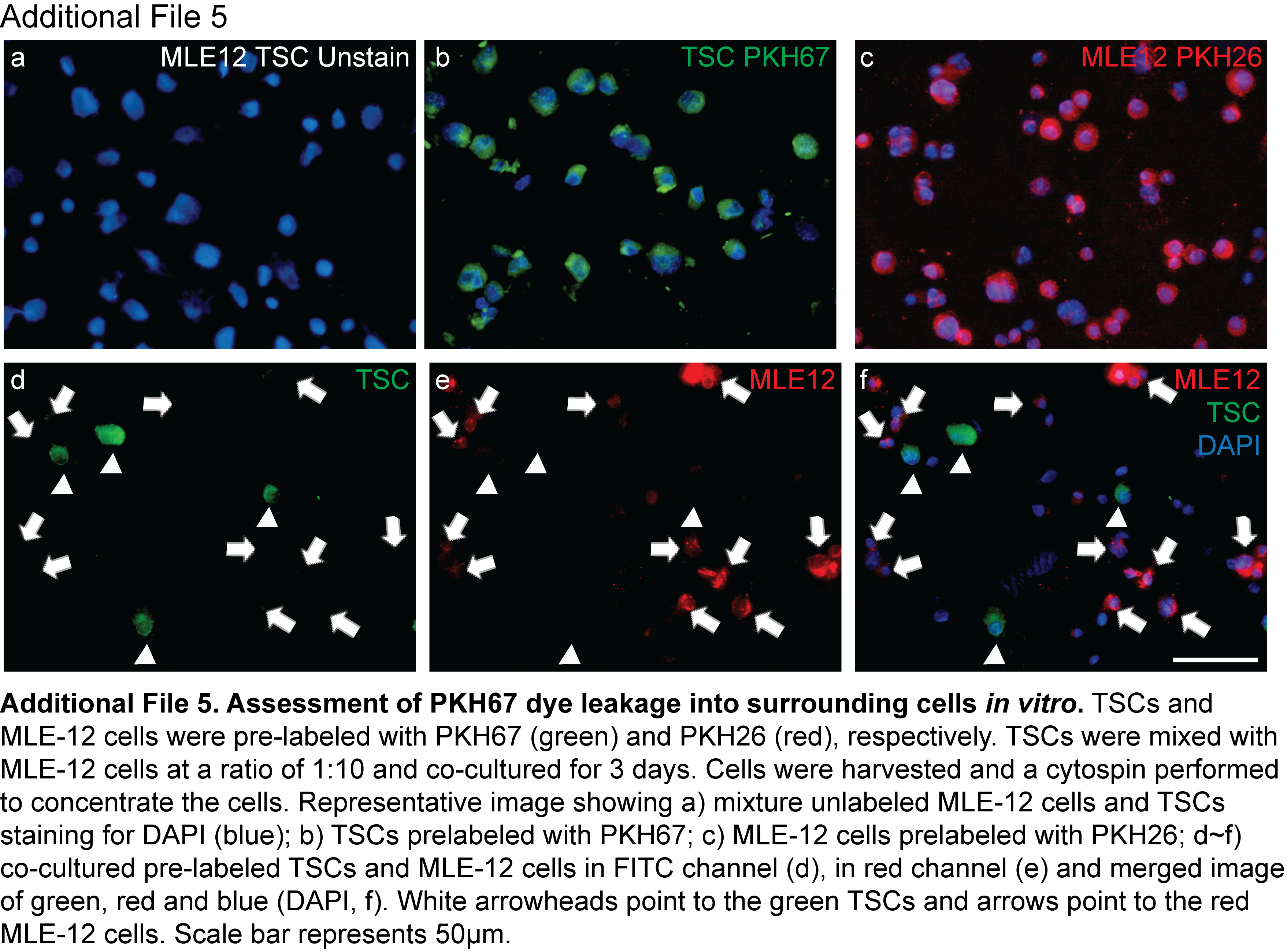

Supplement: Supplementary file 5 — Additional file 5. Assessment of PKH67 dye leakage into surrounding cells in vitro. TSCs and MLE-12 cells were pre-labeled with PKH67 (green) and PKH26 (red), respectively. TSCs were mixed with MLE-12 cells at a ratio of 1:10 and co-cultured for 3 days. Cells were harvested and a cytospin performed to concentrate the cells. Representative image showing a) mixture unlabeled MLE-12 cells and TSCs staining for DAPI (blue); b) TSCs pre-labeled with PKH67; c) MLE-12 cells pre-labeled with PKH26; d~f) co-cultured pre-labeled TSCs and MLE-12 cells in FITC channel (d), in red channel (e) and merged image of green, red and blue (DAPI, f). White arrowheads point to the green TSCs and arrows point to the red MLE-12 cells. Scale bar represents 50µm. [file 13287_2021_2550_MOESM5_ESM.png]
